# Successful Treatment With Lorlatinib Monotherapy for Secondary Central Nervous System Oligometastatic Disease in Refractory Anaplastic Lymphoma Kinase Positive Non-small Cell Lung Cancer

**DOI:** 10.7759/cureus.73645

**Published:** 2024-11-13

**Authors:** Hema Narlapati, Christina Speirs, Ryan M Jones, Jeffrey Berenberg

**Affiliations:** 1 Internal Medicine, Tripler Army Medical Center, Honolulu, USA; 2 Radiation Oncology, Cancer Center of Hawaii, Honolulu, USA; 3 Hematology and Medical Oncology, Tripler Army Medical Center, Honolulu, USA

**Keywords:** alk inhibitor, alk positive lung adenocarcinoma, lorlatinib, metastatic brain tumors, metastatic non-small cell lung cancer, radiation and clinical oncology

## Abstract

The anaplastic lymphoma kinase (ALK) gene plays crucial roles in both normal brain development and oncogenesis, particularly in non-small cell lung cancer (NSCLC). Metastatic ALK-positive NSCLC is characterized by ALK tyrosine kinase domain rearrangements, prompting the use of ALK tyrosine kinase inhibitors (TKIs) to target the mutation. While first-line treatment options include alectinib, brigatinib, and lorlatinib per National Comprehensive Cancer Network (NCCN) guidelines, therapeutic challenges arise in cases of disease progression. Management strategies may involve radiation therapy, switching to alternative ALK inhibitors, or testing for resistance mutations like ALK G1202R to guide treatment selection, with lorlatinib emerging as an alternative treatment option.

Here, we present the case of a 35-year-old male diagnosed with metastatic ALK-positive NSCLC. Despite initial stability on alectinib therapy, disease progression necessitated therapeutic modification, including a switch to brigatinib and subsequent treatment with lorlatinib monotherapy. Notably, the patient achieved complete remission radiologically and clinically following treatment with lorlatinib, highlighting its efficacy in refractory disease settings.

While molecular research supports lorlatinib's superior central nervous system (CNS) penetrability and systemic efficacy, the absence of head-to-head clinical trials presents a significant gap in evidence. Direct comparison of second and third-generation ALK inhibitors is essential to elucidate their comparative efficacy and adverse event profiles, which could refine current management guidelines. Furthermore, if lorlatinib proves superior in terms of progression-free survival, it may offer the potential to delay or obviate the need for radiation therapy, thus mitigating the risk of neurotoxic adverse events associated with these modalities.

## Introduction

The anaplastic lymphoma kinase (ALK) gene plays a crucial role in both normal brain development and oncogenesis, particularly in non-small cell lung cancer (NSCLC), anaplastic large cell lymphoma, and neuroblastoma [1]. In the context of metastatic ALK-positive NSCLC, where the ALK tyrosine kinase domain undergoes rearrangement, ALK tyrosine kinase inhibitors (TKIs) have emerged as a vital therapeutic strategy. These inhibitors target the abnormal ALK protein's ATP pocket, effectively impeding downstream phosphorylation and leading to tumor cell deactivation and death [2].

According to the latest National Comprehensive Cancer Network (NCCN) guidelines, the first-line therapy for treatment, naïve ALK-positive NSCLC typically involves alectinib, brigatinib, or lorlatinib [3]. In cases where patients exhibit disease progression despite initial therapy, options include definitive radiation therapy (RT), switching to another second or third-generation ALK inhibitor, or assessing for specific resistance mutations, such as the ALK G1202R mutation, to guide treatment selection [3].

Clinical trials have demonstrated the efficacy of various ALK inhibitors. The ALEX trial showed that alectinib significantly improved progression-free survival compared to crizotinib in untreated ALK-positive NSCLC patients [4]. Similarly, the CROWN trial revealed that lorlatinib was superior to crizotinib in the first-line treatment of advanced ALK-positive NSCLC [5].

While stereotactic radiosurgery (SRS) offers notable efficacy in achieving high local brain tumor control, it also carries the risk of radiation-induced brain necrosis and edema [6,7]. Given lorlatinib's enhanced ability to penetrate the blood-brain barrier, emerging molecular evidence suggests its potential superiority over second-generation ALK inhibitors, particularly in managing central nervous system metastases [8].

However, despite these promising preclinical findings, the absence of head-to-head trials presents a gap in establishing definitive comparative clinical efficacy between lorlatinib and existing therapeutic options [8]. A recent network meta-analysis attempted to address this gap, comparing lorlatinib, alectinib, and brigatinib in ALK inhibitor-naive and untreated ALK-positive advanced NSCLC, but direct head-to-head trials are needed to solidify these findings that could obviate the need for certain treatment modalities and guide therapeutic management options [9].

While significant progress has been made in the treatment of ALK-positive NSCLC, ongoing research is crucial to optimize treatment strategies and improve patient outcomes. The evolving landscape of ALK inhibitors and their integration with other treatment modalities, such as radiation therapy, presents both challenges and opportunities for enhancing the management of this subset of NSCLC patients.

## Case presentation

**Figure 1:**
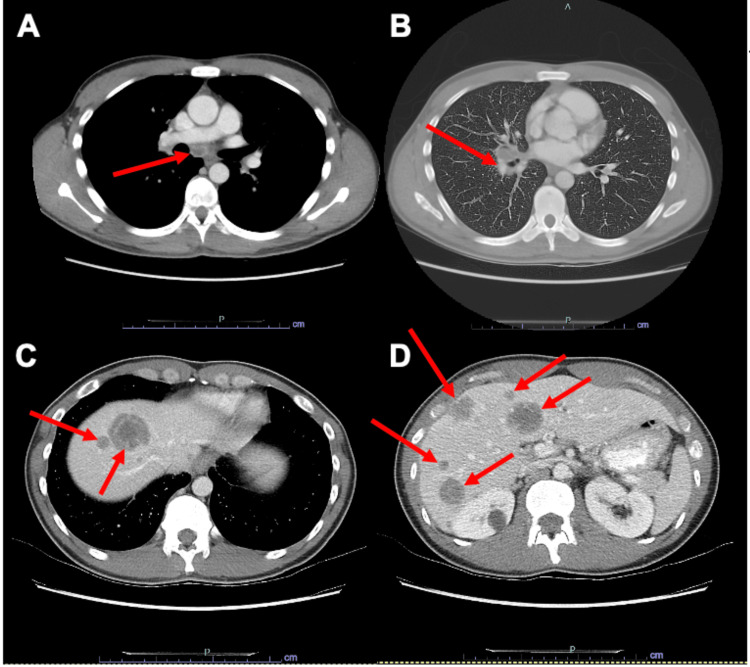
CT axial slices (A) Mediastinal adenopathy. (B) Right perihilar adenopathy. (C, D) Numerous foci of decreased attenuation of varying sizes scattered throughout the liver.

**Figure 2:**
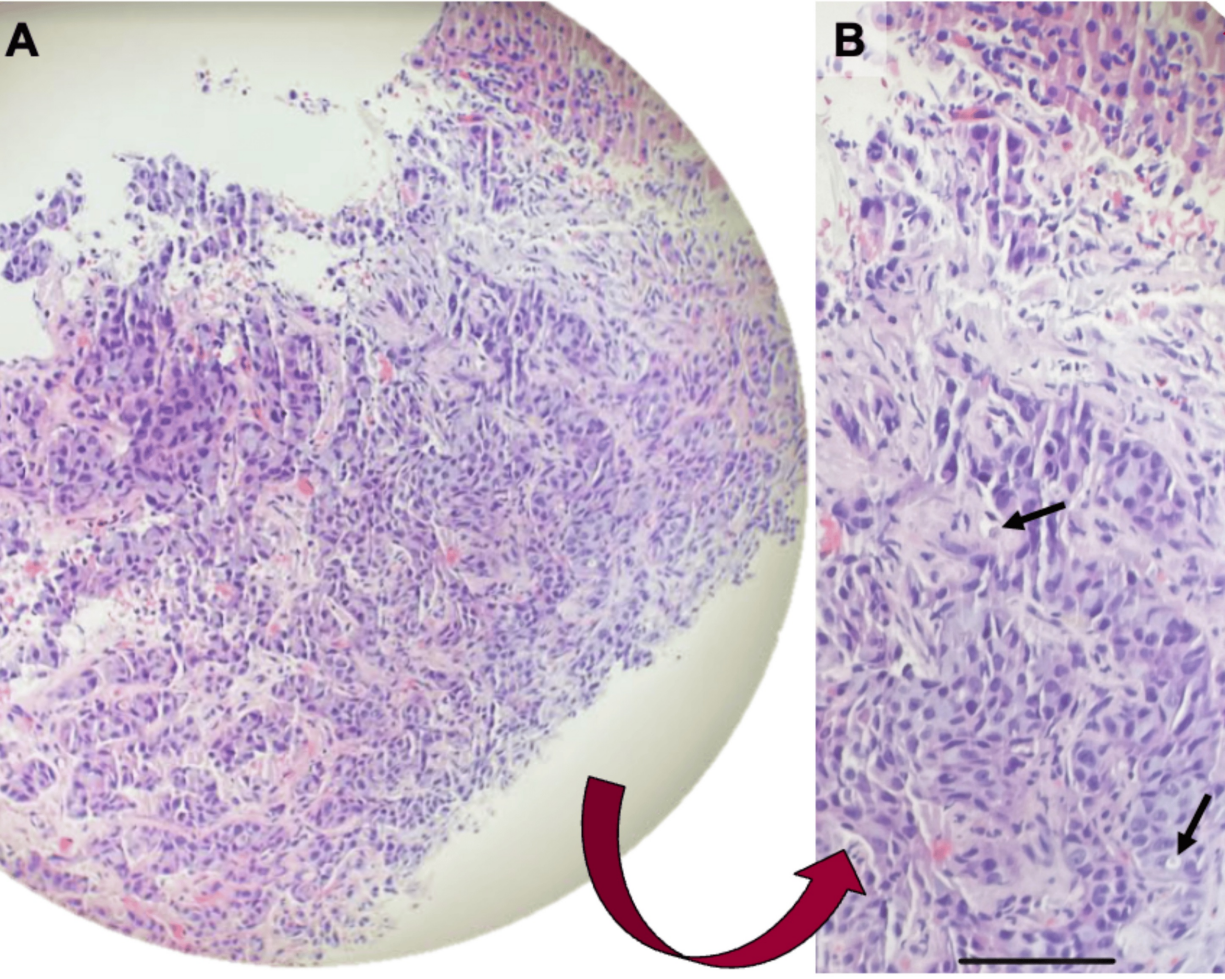
Hematoxylin and eosin stained of liver biopsy (A) Low power view (10X). (B) High power view demonstrating adenocarcinoma with signet ring features (black arrows).

A 35-year-old male presented to Tripler Army Medical Center with a history of chronic cough, persistent bilateral lower chest/rib pain, and unintentional weight loss. Imaging studies, including chest CT and PET/CT scans, revealed the presence of bilateral pulmonary nodules, mediastinal adenopathy, as well as skeletal and liver lesions, suggestive of metastatic disease (Figure 1). Notably, no CNS metastases were evident at this point. A liver lesion biopsy confirmed the presence of adenocarcinoma with signet ring features. Extensive workup, including EGD and colonoscopy, failed to identify a gastrointestinal primary source. However, immunohistochemistry established the diagnosis of NSCLC, specifically the adenocarcinoma subtype (Figure 2), with positive expression for thyroid transcription factor 1 (TTF-1). Genomic testing further unveiled an ALK-positive rearrangement mutation.

**Figure 3:**
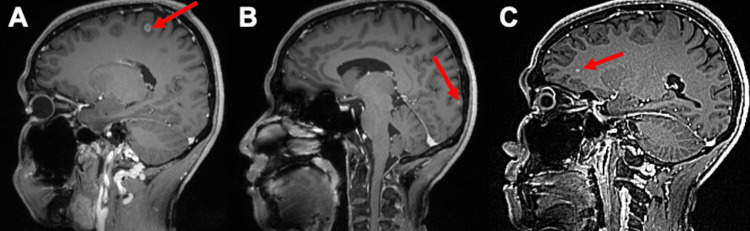
Brain MRI sagittal slices (A, B, C) Multiple small enhancing lesions within the cerebral hemisphere (red arrows).

Following the NCCN guidelines, the patient was started on treatment with alectinib at an initial dose of 600mg orally twice daily. However, due to intolerable adverse effects with myalgias and a marked elevation in CK levels, exceeding ten times the upper limit of normal, the dosage was adjusted to 450mg orally twice daily. The patient exhibited stable disease until surveillance brain MRI revealed new evidence of CNS oligometastatic disease (Figure 3). Consequently, therapeutic management was modified, with the patient transitioning to brigatinib therapy and was referred to the Cancer Center of Hawaii in Honolulu, Hawaii, for SRS with Gamma Knife, based on multidisciplinary team discussion.

In October 2021, due to progressive brain metastasis despite SRS and brigatinib therapy, discussion between radiation oncology and medical oncology initially favored switching to whole brain radiation therapy. However, prior to this decision, the patient was placed on a trial of lorlatinib monotherapy with a halt in the plan for whole-brain radiation therapy after weighing the risks and benefits of a trial of this newer third-generation ALK TKI. Fortunately, a follow-up brain MRI six months later demonstrated marked improvement on lorlatinib monotherapy. Surveillance PET/CT imaging in January 2022 favored the absence of PET avidity of initial liver, skeletal, mediastinal, or pulmonary lesions, favoring treated metastatic disease. This patient continues to remain in both radiological and clinical disease remission, highlighting the efficacy of lorlatinib in this refractory setting.

## Discussion

This case underscores the evolving therapeutic landscape in the management of metastatic ALK-positive NSCLC, with a particular focus on the significance of lorlatinib and its implications for clinical practice and management guidelines.

Lorlatinib, a third-generation ALK inhibitor, has emerged as a promising therapeutic option due to its enhanced blood-brain barrier penetrance and systemic efficacy compared to second-generation counterparts. Recent studies have demonstrated the superior efficacy of lorlatinib over first- and second-generation ALK-TKIs. A matching-adjusted indirect comparison study estimated that lorlatinib improved progression-free survival (PFS) compared to alectinib (HR: 0.54; 95% CI: 0.33, 0.88) and brigatinib (HR: 0.51; 95% CI: 0.31, 0.82) [2]. This aligns with our patient's experience, where lorlatinib demonstrated efficacy after progression on alectinib and brigatinib.

However, the same study also reported a higher rate of Grade ≥3 adverse events with lorlatinib compared to alectinib (RR: 1.48; 95% CI: 1.13, 1.94) [2]. This highlights the need for careful monitoring and management of side effects in patients receiving lorlatinib, a consideration that was not significantly problematic in our case.

CNS metastases represent a significant clinical challenge in ALK-positive NSCLC. While SRS offers localized control, its associated risk of radiation-induced neurotoxicity underscores the importance of exploring alternative treatment modalities. Interestingly, lorlatinib has shown promising results in managing CNS metastases. The aforementioned study estimated that lorlatinib improved time to CNS progression compared with brigatinib (HR: 0.19; 95% CI: 0.05, 0.71) [2]. This is consistent with our patient's experience, where lorlatinib monotherapy led to a marked improvement in brain metastases.

The emergence of resistance mutations, notably ALK G1202R, underscores the necessity of early genetic testing to guide treatment decisions. A network meta-analysis by Wang et al. suggested that lorlatinib might prolong PFS compared to brigatinib (HR: 0.57; p=0.03) and alectinib (HR: 0.65; p=0.05) in previously untreated patients with ALK-positive advanced NSCLC [3]. This supports the potential role of lorlatinib as a first-line treatment option, which could potentially delay the emergence of resistance mutations.

While molecular evidence suggests the potential superiority of lorlatinib in ALK-positive NSCLC, further clinical research is warranted to validate its efficacy and safety profile. The lack of head-to-head trials comparing different generations of ALK inhibitors remains a significant gap in the evidence base [3]. Our case report adds to the growing body of real-world evidence supporting the efficacy of lorlatinib in refractory settings, but randomized controlled trials are needed to definitively establish its place in treatment algorithms.

Should lorlatinib demonstrate clinical superiority in terms of progression-free survival in head-to-head trials, its incorporation into clinical practice may lead to paradigm shifts in treatment algorithms. Specifically, the potential to delay or even obviate the need for radiation therapy, including stereotactic radiosurgery or whole-brain radiation therapy, could significantly mitigate the risk of neurotoxic adverse events associated with these modalities. This aligns with our patient's experience, where lorlatinib monotherapy achieved disease control without the need for whole-brain radiation therapy.

Furthermore, while our case report and recent studies suggest promising outcomes with lorlatinib, particularly in managing CNS metastases and overcoming resistance to earlier-generation ALK inhibitors, more robust clinical evidence is needed to optimize treatment strategies and improve patient outcomes in ALK-positive NSCLC.

## Conclusions

While molecular research suggests improved CNS penetrability and systemic efficacy of third-generation ALK inhibitors, robust evidence from head-to-head clinical trials is needed to translate into the clinical setting to demonstrate superiority. A comparison of second and third-generation ALK inhibitors is crucial to question treatment options and refine management guidelines. Additionally, further clinical research is warranted to validate the efficacy and safety profile of third-generation ALK inhibitors compared to second-generation ALK inhibitors. If lorlatinib proves superiority in progression-free survival over second-generation ALK inhibitors, this may revolutionize treatment algorithms, potentially reducing the need for radiation therapy and mitigating associated neurotoxic adverse events. Moreover, the emergence of resistance mutations highlights the importance of early genetic testing if there is a concern for the development of resistance mutations to second-generation ALK inhibitors, for which lorlatinib can be used. Further clinical research is warranted to validate lorlatinib's efficacy and safety profile, thereby advancing personalized treatment options and improving outcomes for patients with ALK-positive non-small cell lung cancer.
